# Atherogenic index of plasma as a predictor of coronary artery disease: a cohort study in south of Iran

**DOI:** 10.1186/s43044-024-00497-z

**Published:** 2024-05-28

**Authors:** Mahdieh Mirzadeh, Marzieh Nikparvar, Shideh Rafati, Masoumeh Kheirandish, Abnoos Azarbad, MohammadHosein Sheybani-Arani, Fatemeh Khajavi-Mayvan, Reza Morovatshoar

**Affiliations:** 1 Clinical Research Development Center of Shahid Mohammadi Hospital, Hormozgan University of Medical Sciences, Bandar Abbas, Iran; 2 Endocrinology and Metabolism Research Center, Hormozgan University of Medical Sciences, Bandar Abbas, Iran; 3 Social Determinants in Health Promotion Research Center, Hormozgan University of Medical Sciences, Bandar Abbas, Iran

**Keywords:** Coronary artery disease, Atherogenic index of plasma, PERSIAN cohort

## Abstract

**Background:**

Coronary artery disease (CAD) is an atherosclerotic disease of an inflammatory nature. Previous studies examining the relationship between triglycerides and high-density lipoprotein cholesterol have highlighted the importance of plasma atherogenic index (AIP) as an important predictor of coronary heart disease. However, due to the lack of adequate information on this topic, this study aimed to investigate the relationship between AIP and coronary heart disease risk.

**Results:**

This study included 2,226 women and 1,690 men aged 35–70 years who participated in the Bandar Kong Cohort study and met the eligibility criteria. The data was collected using a checklist and questionnaires, which were designed by experienced individuals. After participants completed a registration form and gave informed consent, face-to-face interviews were conducted by trained experts. The validity and reliability of the questionnaire had been verified by the national cohort team prior to its use. The Ethics Committee of Hormozgan University of Medical Sciences (IR.HUMS.REC.1400.171) approved the study. Data from the initial cohort survey using SPSS software version 25, were analyzed to include several factors, including age, sex, smoking status, body mass index (BMI), physical activity level, socioeconomic status, AIP, systolic blood pressure, and diastolic blood pressure. The prevalence of coronary heart disease was found to be 7.5% higher in people with a BMI of 25 or higher. Also, Individuals with low physical activity had a higher prevalence. Individuals with CAD had significantly higher mean values for the AIP, age, systolic blood pressure, and diastolic blood pressure (0.46, 57.50, 128.43, and 81.10, respectively) compared to those without CAD. Furthermore, patients with CAD had lower years of education (2649.45 and 3.59) than individuals without CAD (*P* < 0.05). Importantly, our findings showed that AIP increased the odds ratio of coronary heart disease by 1.86 as an independent risk factor.

**Conclusions:**

Based on our investigation, the AIP is a valuable and independent predictive risk factor for coronary artery disease. This index can be utilized effectively due to its accessibility and affordability, making it a promising tool for risk assessment in clinical settings.

## Background

CVDs are the first cause of death globally. In 2015 alone, about 17.9 million individuals died of CVDs. Stroke and ischemic heart disease (IHD) were the primary contributors to cardiovascular-related health loss in all regions [1]. Given the substantial economic burden associated with CVDs, early identification of major risk factors is crucial to implementing preventive strategies promptly [2].

Coronary artery disease (CAD) is a cardiovascular disorder that occurs due to atherosclerosis or atherosclerotic blockage of coronary arteries [3]. When the endothelial function of the arterial wall is disrupted, atherosclerosis begins due to the accumulation of lipoprotein droplets in the intima of the coronary arteries [4]. In the bloodstream, water-insoluble lipids circulate by attaching themselves to water-soluble lipoproteins called apolipoproteins. Low-density lipoproteins (LDL) in high concentrations can penetrate the disturbed endothelium and undergo oxidation [5]. This oxidized or modified LDL then attracts leukocytes into the intima of coronary vessels, which are cleared by macrophages and lead to the formation of foamy cells. These foam cells multiply and form lesions called sebaceous glands. The formation of such lesions creates signals that attract smooth muscle cells (SMCs) to the location of the fatty vein. Then SMCs start to multiply and produce extracellular matrix, mainly collagen and proteoglycans. Atherosclerotic plaque begins to grow and accumulate a large volume of extracellular matrix produced by SMCs and leads to the progression of the lesion to fibrous plaque. Fibrous plaque penetrates the channels of coronary arteries and small blood vessels that were formed and can subsequently calcify the plaques. The final lesion formed is an advanced and complex lesion consisting of a fibrous cap with a fat-rich core containing necrotic material that may be highly thrombogenic [6].

As a result of the formation of atherosclerotic plaque in the coronary artery, blood flow is blocked, which leads to a mismatch between myocardial oxygen demand and supply [7].

Lipid profile includes a group of biochemical tests that are often used in the prediction, diagnosis, and treatment of lipid-related disorders, including arteriosclerosis. In general, blood lipids are considered risk factors for IHD and peripheral vessels. A strong correlation between the risk of CAD, high LDL-C level, and low HDL-C level is well established. However, the huge role of triglycerides (TGs) in cardiovascular risk has been underestimated. High levels of triglycerides are associated with an increase in the incidence of CAD and an increase in the population of small dense LDL-C particles [8].

High-density lipoprotein cholesterol’s (HDL-C) relationship with TGs has been explored by numerous studies; revealing that the TG to HDL-C ratio serves as a predictive indicator for myocardial infarction. Some researchers have globally utilized the log (TG/HDL-C), atherogenic index of plasma (AIP), as a key predictor for atherosclerosis [8]. AIP is based on two important parameters, TG and HDLc, both of which are independent risk factors for CAD [9]. AIP can be a diagnostic tool when other atherogenic risk parameters appear within normal ranges [10]. However, no prior study has examined the relationship between CAD risk and AIP within the Bandar Kong population; Hence, our objective is to investigate this matter.

## Methods

### Participants and design

This cohort study is carried out using the data of a primary survey in the Bandar Kong cohort study [11]; which is part of a large Persian multicenter study [12] on 4063 individuals aged 35–70 years residing in Bandar Kong. The cohort center was set up with carefully designed spaces for various activities such as reception, enrollment, sample collection, measurements, and interviews. Additionally, it had a fully equipped laboratory with a standard biobank. All the necessary equipment and supplies were procured and arranged by qualified professionals according to the PERSIAN Cohort Protocol. For more information, please refer to the cited articles.

The main phase of the Bandar Kong study started on Oct. 27, 2016. The study aimed to recruit 4200 participants from a population of 6000 permanent residents aged 35–70 years. The participants were selected based on a sample size calculation using the Fleiss formula, considering factors such as the desired power, significance level, and the ratio of sample size for exposed and unexposed groups. The study employed a local multidisciplinary team of researchers, including a physician, six interviewers, a nurse, an administration support officer, a field manager, an epidemiologist, two nutritionists, and a biochemist to collect data. Interviewers visited each household, explained the study, and provided a pamphlet with further details. After obtaining informed consent, each participant received a dated invitation and was reminded by phone one week and one day before the scheduled event. The data collection process involved a comprehensive set of measurements, including blood samples, hair and nail clippings, anthropometric measurements, and a detailed interview. The study also included a follow-up phase, where participants were re-interviewed every 5 years, with annual check-ups, to monitor changes in lifestyle and health outcomes. The data collected was reviewed by medical consultants to assign diagnosis codes and track the development of chronic NCDs over time.

Data related to CAD and confounding factors such as age, sex, body mass index (BMI), socioeconomic status, education level, physical activity, history of chronic diseases, and other factors were extracted from the Bandar Kong project data. For this study, we included all eligible individuals (3916 participants) from the Bandar Kong cohort who met the inclusion criteria and did not meet the exclusion criteria. The participants were randomly selected using a census sampling method.

### Inclusion and exclusion criteria

The inclusion criteria for this study were individuals with CAD who self-reported or had clinical medical records. CAD is a condition characterized by the narrowing or blockage of the coronary arteries, which are responsible for delivering blood and oxygen to the heart [13]. Pregnant women, individuals with acute underlying diseases (in order to exclude items distorting our results, affecting the outcome, and avoiding unwanted complications), participants who withdrew or were reluctant, those whose medical records weren’t sufficiently completed to join the study, and the outliers(those whose data could influence the confidence interval, distort our mean value and raise the variance) were excluded from the study.

### Ethical considerations

The information obtained during the Bandar Kong cohort study was utilized after obtaining research approval and ethical clearance from the Ethics Committee of Hormozgan University of Medical Sciences (IR.HUMS.REC.1400.171). All participants consented to participate prior to their participation. Informed written consent to participate in the study was provided by all participants (or their parents or legal guardians in the case of children under 16). Consent for publication was obtained from all participants.

### Measurement and definitions

All the required information, including demographic information and desired data, was collected using a checklist prepared by the researcher. Participants' information was collected through questionnaires designed by fully trained and experienced individuals following the completion of the registration form and informed consent. The data collection process involved face-to-face interviews conducted by trained experts at the location of the cohort study. The questionnaire covered various aspects such as general characteristics, medical examination, blood pressure measurement, socioeconomic status, employment records, living conditions, lifestyle, medical history, medication usage, family records, sleep patterns, physical activity, personal habits, food consumption, dietary supplements, and dietary habits. As the Bandar Kong cohort study is part of the PERSIAN national cohort study, the validity and reliability of the questionnaire had been verified by the national cohort team before implementation.

#### Anthropometric indicators

Weight without shoes and minimal clothing was measured and recorded by trained personnel using a scale with 100 g accuracy. Height was measured using a 206 Seka wall-mounted height meter, with individuals standing without shoes against the wall, shoulders in their usual position, and measurements taken with one-centimeter accuracy.

#### Other variables

Additional variables such as age, gender, socioeconomic status, education level, coffee consumption, history of chronic diseases (diabetes, CVD, blood pressure, cancer, liver, kidney, and digestive diseases, depression), and the amount of time spent using television and computers were obtained as primary information from all participants in the Bandar Kong cohort.

#### Blood pressure assessment

Blood pressure was measured while the participants were seated and at rest for 15 min using a standard and calibrated mercury sphygmomanometer. Two measurements were taken from both arms at a 10-min interval, and the average value was recorded. Individuals with an average blood pressure of 140/90 mm Hg or higher or those under medical treatment for hypertension were considered to have hypertension.

#### Level of physical activity

Participants were asked to define a typical working day, and if they engaged in activities that did not occur daily, the monthly frequency was considered until it equated to an entire 24-h day. They were then asked to recall their daily activities and provide an average representation of their activity levels over the past year. Each physical activity was assigned a metabolic equivalent (MET) value, indicating the amount of energy expended per kilogram of body weight per hour. The time spent on different activities with varying intensities was recorded, multiplied by the respective MET values, and then adjusted for the number of days the activity was repeated. The sum of these values provided each individual's MET hours per day.

#### Diet survey

Data regarding participants' food intake was collected through a face-to-face interview using a food frequency questionnaire (FFQ). The FFQ used in the Bandar Kong cohort study required individuals to report the frequency and quantity of food consumed per day, week, and month.

#### Experiments

Blood samples were collected from each participant after a fasting period of 10 to 12 h to measure fasting blood sugar and lipid profile.

### Statistical analysis

SPSS software, version 25 used for statistical analysis. The Kolmogorov–Smirnov test was employed to assess the normal distribution of the data. Descriptive statistics were used to estimate the prevalence of CAD. The Chi-Square test was used to compare the relationship between categorical variables among different groups, while the T-test was utilized to compare the means of quantitative variables between different groups. In cases where variables did not follow a normal distribution, the Mann–Whitney test was used. Logistic regression was employed, both in crude and multivariable models, to assess the simultaneous effects of variables on coronary heart disease. A p-value less than 0.05 was considered statistically significant.

## Results

Three thousand, nine hundred and sixteen participants were included in this study, of whom 2,226 (56.8%) were women and 1,690 (43.2%) were men. According to the reported results, the prevalence of self-reported CAD among the participants was 8.7% (341 people). The prevalence of CAD in people with a BMI greater than or equal to 25 (9.4%) was higher than in those with a BMI less than 25 (7.5%). There was a higher prevalence of CAD among economically poor individuals (10.0%) than among those of medium economic status (8.5%) and wealthy individuals (7.5%). Individuals with low physical activity had a prevalence of 14.7%, while those with moderate physical activity had a prevalence of 6.6%, and those with high physical activity had a lower prevalence (5.8%). The prevalence of CAD was 12% in smokers and 8% in non-smokers (Table 1).

**Table 1:** General characteristics of study participants with/without coronary artery disease

Variables	Coronary Artery Disease (CAD)				*P*-value*
	Non-CAD (*n* = 3575)		CAD (*n* = 341)		
	Frequency	Percentage (%)	Frequency	Percentage (%)	
Gender					
Men	1533	90.7	157	9.3	0.26
Women	2042	91.7	184	8.3	
BMI (kg/m^2^)					
<25	1328	92.5	107	7.5	0.03
≥25	2247	90.6	234	9.4	
Economic status					
Low	1442	90.0	160	10.0	0.04
Middle	689	91.5	64	8.5	
High	1444	92.5	117	7.5	
Physical activity METS/daily					
24–36/5	861	85.3	148	14.7	< 0.001
36/6–44/9	2135	93.4	152	6.6	
≤45	533	94.2	33	5.8	
Smoking					
No	3062	92	268	8	< 0.002
Yes	501	88	68	12	

*The Chi-Square test is used to obtain the written *P* values

Based on the results of independent T-tests, individuals with CAD had significantly higher mean values for the AIP, age, systolic blood pressure, and diastolic blood pressure (0.46, 57.50, 128.43, and 81.10, respectively) compared to those without CAD. Additionally, patients with CAD had lower daily energy intake and fewer years of education (2649.45 and 3.59) than individuals without CAD showing a statistically significant relationship (*P* < 0.05) (Table 2).

**Table 2:** The relationship between the average quantitative variables of the studied subjects and coronary artery disease

Variables	Coronary artery disease (CAD)		*P*-value*
	Non-CAD	CAD	
	Mean ± SD**	Mean ± SD	
Atherogenic Index of Plasma	0.40 ± 0.26	0.46 ± 0.26	< 0.001
TG	135.61 ± 88.69	147.31 ± 81.67	< 0.001
HDLc	48.05 ± 10.78	45.57 ± 9.81	< 0.001
LDL-C	129.22 ± 33.47	110.27 ± 40.83	< 0.001
Age (years)	47.41 ± 9.03	57.51 ± 7.99	< 0.001
Systolic blood pressure < 0/001 (mmHg)	117.76 ± 16.78	128.43 ± 21.02	< 0.001
Diastolic blood pressure (mmHg)	76.56 ± 10.24	81.10 ± 10.70	< 0.001
Daily energy intake	2919.93 ± 43.53	2649.89 ± 45.72	< 0.001
Years of education	6.4 ± 3.81	3.4 ± 59.14	< 0.001

*Mann–Whitney and T-tests are used to obtain the written *P*-Values

**SD Standard deviation

Table 3 presents the estimation of coefficients, odds ratio (OR), 95% confidence interval (CI), and *P*-value of the univariate and multivariate models using logistic regression to predict the presence of CAD using the AIP.

**Table 3:** Logistic regression analysis to predict coronary artery disease using plasma atherogenic index

Variable	Crude					Adjusted				
	*B*	OR	Confidence interval 95%		*P* value*	*B*	OR**	Confidence interval 95%		*P* value*
			Lower bound	Upper bound				Lower bound	Upper bound	
API	0.86	2.35	1.56	3.55	<0.001	0.62	1.86	1.12	3.08	0.02
Age (years)	0.12	1.12	1.11	1.14	<0.001	0.09	1.09	1.07	1.11	<0.001
Years of education	−0.13	0.87	0.85	0.90	<0.001	0.005	1.005	0.97	1.04	0.78
Daily Energy Intake	0.00	1.00	1.00	1.00	<0.001	0.00	1.00	1.00	1.00	0.04
Gender										
Men	0.12	1.13	0.90	1.42	0.26	–	–	–	–	–
Woman	–	1	–	–	–	–	–	–	–	–
Hypertension										
No	–	1	–	–	–	–	1	–	–	–
Yes	203	7.60	5.88	9.81	<0.001	1.33	3.80	2.86	5.05	<0.001
BMI (kg/m^2^)										
< 25	–	1	–	–	–	–	1	–	–	–
≥ 25	0.26	1.29	1.02	1.64	0.03	0.19	1.21	0.91	1.59	0.18
Economic Status										
Low	0.31	1.37	1.07	1.76	0.01	0.02	1.02	0.75	1.39	0.9
Middle	0.14	1.15	0.83	1.57	0.40	0.04	1.05	0.73	1.51	0.81
High	–	1	–	–	–	–	1	–	–	–
Physical activity METS/daily										
24–36/5	1.02	2.77	1.87	4.11	0.00	0.46	1.58	1.03	2.42	0.04
44/9–36/6	0.14	1.15	0.78	1.69	0.48	− 0.02	0.98	0.65	1.48	0.92
≥ 45	–	1	–	–	–	–	1	–	–	–
Smoking										
Yes	0.44	1.55	1.17	2.06	0.002	0.38	1.46	1.04	2.03	0.03
No	–	1	–	–	–	–	1	–	–	–

*Multivariate logistic analysis is used to obtain the written *P*-values

**OR Odds ratio

The multivariate logistic analysis revealed that the AIP, considering other risk factors for CAD such as age, hypertension, daily energy intake, physical activity, and smoking as independent variables, predicted approximately 86% increase in the likelihood of developing CAD (OR = 1.86; 95% CI 1.12, 3.08).

## Discussion

CAD is a highly prevalent and incident condition and is associated with one of the highest mortality rates in the world [14, 15]. Myocardial infarction, sudden cardiac death, unstable or stable angina, and myocardial infarction are the symptoms of CAD, an inflammatory atherosclerotic disease [16]. Significant risk factors for CAD include non-modifiable factors like age and modifiable factors like high blood pressure, high cholesterol, smoking, diabetes, obesity, or overweight, physical inactivity, poor eating habits, and stress. Age, gender (men are more likely than women to develop CAD), race, and family history all increase the risk.

In this study, we analyzed risk factors for CAD based on the AIP using population data from the Bandar Kong cohort. AIP, calculated as log10 (TG/HDL-C), was originally developed as a plasma biomarker of atherosclerosis. The value is used to calculate small dense LDL (sdLDL). [17]. With its high LDL content and small size, sdLDL is sensitive to oxidative stress and is easily converted to oxidized LDL in the body. This process causes an inflammatory response in the subendothelial layer of blood vessels and triggers the production of foam cells, contributing to the development of atherosclerosis [18]. However, the analysis of sdLDL has been challenging due to its cost and complex process.

In comparison, AIP provides an easily obtainable and accurate reflection of sdLDL. As a result, it has emerged as a predictive index for CAD. It has been proposed that an AIP value less than 0.11 is related to a low CVD risk, while values between 0.11 and 0.21 and above 0.21 indicate moderate and increased risks, respectively [19].

This research demonstrated AIP, considering other risk factors such as age, hypertension, daily energy intake, physical activity, and smoking, predicted an approximately 86% increase in the incidence of CAD in the Bandar Kong cohort population. A study by Yung LY et al. demonstrated a connection between AIP and obesity, waist circumference, blood sugar, and blood lipid profile in the Korean male population, suggesting the predictive potential of AIP for CAD [20]. Zhu et al. [21] and Kim [22] conducted studies where AIP values were divided into quartiles and found that BMI values increased as AIP values increased from the first to fourth quartiles. Specifically, Zhu et al. established a link between AIP and obesity, with higher AIP levels strongly associated with obesity when a BMI greater than or equal to 28 kg/m2 was defined as obesity [21]. Shen et al. examined the correlation between AIP and waist circumference, demonstrating that AIP values between 0.12 and 0.21 or above 0.21 indicate the possibility of borderline abdominal obesity and abdominal obesity, respectively. Thus, AIP can serve as an estimation for abdominal obesity [23].

In our study, AIP was found to increase the risk of CAD, taking into account smoking and physical activity. Yung Ly et al. [20] also observed a significant relationship between AIP, physical activity, and smoking. Reduced physical activity [24] and excessive smoking [25, 26] can lead to elevated blood TG levels.

Numerous previous studies have established a positive relationship between AIP and TG, total cholesterol (TC), and low-density lipoprotein-cholesterol (LDL-C), as well as a negative relationship with HDL-C [10, 27, 28]. While these lipid components have traditionally been used to predict atherosclerotic CVDs, AIP offers the advantage of being a more relevant biochemical parameter than the conventional atherogenic index (AI) [29].

The study's primary strengths lie in its excellent design, relatively large sample size, and the accurate measurement of anthropometric parameters and blood pressure by a uniquely trained research team operating in the coastal region of southern Iran. Therefore, the information on NCDs and their risk factors gathered from this study can provide valuable insights into prevention and management strategies. However, there are several limitations to consider. The data used in this study were extracted from a cohort study conducted among Iranians in the south of the country, specifically aged 35–70 years, making it a unique study population. Whether the findings can be generalized to other age groups or ethnicities remains unclear. Additionally, the study could not assess a longitudinal relationship between the AIP and CAD. Our study would have benefitted from a classification of CAD based on AIP ranges and its relation with the risk of CAD which should be considered in future studies. Also, a more comprehensive dietary data would result in better interpreted results which shouldn’t go unnoticed.

## Conclusions

The findings from this study demonstrate that the AIP holds promise as an independent predictive risk factor for CAD, offering a cost-effective and readily available tool in medicine. Nonetheless, it is advisable to validate these results further through longitudinal and prospective studies.

## Data Availability

The data sets used during the current study are available from the corresponding author upon reasonable request.

## Abbreviations

AIP: Atherogenic index of plasma
BMI: Body mass index
CVDs: Cardiovascular diseases
IHD: Ischemic heart disease
HDL-C: High-density lipoprotein cholesterol
CAD: Coronary artery disease
MET: Metabolic equivalent
FFQ: Food frequency questionnaire
OR: Odds ratio
CI: Confidence interval
sdLDL: Small dense LDL
TG: Triglycerides
AI: Atherogenic index
TC: Total cholesterol
LDL-C: Low-density lipoprotein-cholesterol
